# A Case-Control Study Exploring the Role of Serum Manganese Superoxide Dismutase (MnSOD) Levels in Gastric Cancer

**DOI:** 10.2188/jea.15.90

**Published:** 2005-06-01

**Authors:** Yingsong Lin, Shogo Kikuchi, Yuki Obata, Kiyoko Yagyu, the Tokyo Research Group on Prevention of Gastric Cancer

**Affiliations:** 1 Department of Public Health, Aichi Medical University School of Medicine.

**Keywords:** Manganese, serum MnSOD levels, Stomach Neoplasms, Risk, Case-Control Studies

## Abstract

BACKGROUND: The role of serum manganese superoxide dismutase (MnSOD) in the development of gastric cancer has not been clearly defined.

METHODS: We conducted a case-control study to address the potential relationship between serum MnSOD levels and gastric cancer. Cases were 275 gastric cancer patients and controls were 275 sex- and age-matched healthy persons. Serum MnSOD levels were determined by a commercially available enzyme-linked immunosorbent assay (ELISA).

RESULTS: The mean(±standard deviation) of serum MnSOD levels was 177.4±87.3 ng/mL among cases and 169.4±56.7 ng/mL among controls. Gastric cancer patients had slightly higher serum MnSOD levels than the controls. After adjustment for pack-years of cigarette smoking and *Helicobacter pylori* infection, the odds ratio was 1.54(95% confidence interval; 0.79-3.01) for subjects in the highest quartile versus the lowest quartile. No significant differences were observed for serum MnSOD levels in gastric cancer patients according to clinicopathological factors such as disease stage, histological type, venous invasion, and lymph node metastasis.

CONCLUSION: Our study suggested that serum MnSOD levels are not significantly associated with the increased risk of gastric cancer, although a weak association may exist.

Gastric cancer is a disease of complex etiology involving multiple risk factors including dietary, infectious, occupational, genetic, and preneoplastic factors, and remains one of the major health burden in Japan. Gastric carcinogenesis may be attributed in part to reactive oxygen species, which has been shown to participate in the multistage carcinogenesis from initiation to malignant conversion by causing oxidative DNA damage and mutations in proto-oncogenes and tumor suppressor genes, and by activating signal transduction pathways.^1^ In response to oxidative stress, the body has formed an elaborate antioxidant system to detoxificate reactive oxygen species. In this antioxidant system, manganese superoxide dismutase (MnSOD) is the major antioxidant that converts superoxide radical to hydrogen peroxide and molecular oxygen within mitochondrial matrix. Previous studies on MnSOD expression in cancer were mainly based on the small number of tumor tissues or cell lines, and the findings have been conflicting.^2^^-^^4^ Few studies, however, have examined serum MnSOD levels in a large number of patients with gastric cancer and apparently healthy persons.

We sought to evaluate the potential relationship between serum MnSOD levels and the risk of gastric cancer in a case-control study.

## METHODS

Case subjects were patients who were newly diagnosed with primary gastric cancer at one of nine hospitals in the Tokyo Metropolitan Area between 1993 and 1995. Patients were included into our study if they were 20-69 years of age and did not undergo surgical treatment for gastric cancer. An endoscopy was performed on all eligible cases and the diagnosis was confirmed by an examination of the resection or biopsy specimen. Data on pathological findings, including the type and stage of the cancer, were recorded for each case. Gastric cancer was further categorized into early and advanced cancer, as well as intestinal and diffuse type, based on the criteria proposed by the Japanese Research Society for Gastric Cancer.^5^ Control subjects were recruited from apparently healthy persons who underwent medical checkups at a health promotion center in the same area. Written informed consent was obtained form each of the study participants before he or she provided a blood sample. Besides, both case and control subjects were asked to fill out a questionnaire regarding smoking and drinking habits. Information on smoking included smoking status (nonsmoker, ex-smoker, or current smoker), the number of cigarettes smoked per day, and years of smoking. Between 1993 and 1995, we enrolled 788 gastric cancer patients and 1,007 apparently healthy controls. Since our study is an exploratory investigation, we selected 275 gastric cancer patients to form the case group. The process of selection is as follows: first, because of the small number of gastric cancer patients under 40 years of age, we selected the majority of them in this age group. Second, in consideration of sex and age distribution, we randomly selected the other patients in each 10-year age group. For each case subject, one control subject was selected at random and matched for case on sex and age(±2 years).

Serum samples of cases and controls were collected in the same method and were frozen at -80°C until analysis. Serum levels of MnSOD were determined by a commercially available enzyme-linked immunosorbent assay (ELISA, Amersham,Pharmacia Biotech, NJ, USA) using the manufacturer’s assay procedure. During measurement, serum samples were analyzed in randomly ordered duplicates in order to reduce systematic and interassay error. Sera were also analyzed for H. pylori immunoglobulin G antibodies by ELISA kits (J-HM-CAP, Kyowa Medex, Japan). The cut-off value for *Helicobacter pylori* infection was 2.7 ELISA value (EV). Additionally, serum pepsinogen was measured using radioimmunoassay (RIA), with pepsinogen I ≤ 70 ng/mL and pepsinogen I/II ≤ 3 indicating atrophy of gastric mucosa. All the assays were carried out and interpreted by individuals who were blinded to the case-control status of samples.

The differences in means between case subjects and control subjects were tested by t-test or Wilcoxon’s rank sum tests. Differences in percentages were tested by Chi-square test. Serum MnSOD levels were categorize into quartiles on the basis of their distributions among the controls. Odds ratios (ORs) and 95% confidence intervals (CIs) from conditional logistic regression model were used to evaluate the strength of the association. The ORs were presented with the lowest quartile being the reference category, and were adjusted for potential confounding factors such as pack-year of smoking and H. pylori infection. To test for a linear trend across the quartiles, we coded each quartile as 0, 1, 2, and 3, and then incorporated this data into the logistic model as a single variable. All analyses were conducted using SAS^®^ release 6.12 (SAS institute, Inc., Cary, NC).

The study was approved by the Ethics Board of Aichi Medical University School of Medicine.

## RESULTS

The mean age ± standard deviation was 53.5 ± 10.5 years in cases, and 53.6 ± 10.5 years in controls (Table 1). The range of serum MnSOD levels was 0-1013.5 ng/mL among case subjects and 0-442.5 ng/mL among control subjects. The mean(± standard deviation) of serum MnSOD levels was 177.4 ± 87.3 ng/mL in case subjects, as compared with 169.4 ± 56.7 ng/mL among control subjects. Cases subjects had a statistically marginal increase in serum MnSOD levels than control subjects (p=0.09). Ninety-four percent of case subjects and 62% of control subjects had *H. pylori* infection (Table 1).

**Table 1.** Characterisitics of the Study Subjects.

		Cases (n=275)		Controls (n=275)		P for difference
	
		N	(%)	N	(%)	
Sex						
	Men	142	(51.6)	142	(51.6)	
	Women	133	(48.4)	133	(48.4)	

Age (mean ± standard deviation)		53.5 ± 10.5		53.6 ± 10.5		

MnSOD* level (mean ± standard deviation)		177.4 ± 87.3		169.4 ± 56.7		0.09

Cigarette smoking						
	Nonsmokers	127	(46.5)	141	(52.4)	0.17
	Smokers	146	(53.5)	128	(47.6)	

*Helicobacter pylori* infection						<0.01
	Negative	18	(6.5)	105	(38.2)	
	Positive	257	(93.5)	170	(61.8)	

Atrophy of gastric mucosa						
	Yes	156	(56.7)	96	(34.9)	<0.01
	No	119	(43.3)	179	(65.1)	

*: Serum manganese superoxide dismutase.

Cases had a higher proportion of atrophy of gastric mucosa, but the mean of serum MnSOD levels did not differ significantly among controls with or without atrophy of gastric mucosa. Among control subjects, the mean of serum MnSOD levels was significantly higher in men (176 ± 56.5 ng/mL) than in women (159.0 ± 55.2 ng/mL, p=0.002). This result was also observed for each age group (Table 2). The mean of serum MnSOD levels did not differ significantly between those with H. pylori infection (174.3±5.02 ng/ml) and those without *H. pylori* infection (166.9 ± 60.4 ng/mL).

**Table 2.** Serum manganese superoxide dismutase (MnSOD) levels (mean ± standard deviation) by sex and age among controls.

Age (year)	Men (N=142)	Women (N=133)
20-39	182.6±35.1 (13)	148.0±29.8 (19)
40-49	183.0±56.8 (31)	155.6±39.4 (27)
50-59	175.7±51.9 (48)	151.6±52.3 (43)
60-69	180.8±65.5 (50)	173.0±71.0 (44)

No. of subjects in parentheses.

Table 3 presents ORs for gastric cancer according to serum MnSOD levels. After adjustment for pack-years of smoking and *H. pylori* infection, the OR for subjects in the highest quartile versus the lowest quartile was 1.54 (95% CI; 0.79-3.01).

**Table 3.** Odds ratios (Ors) for gastric cancer according to quartile of serum manganese superoxide dismutase (MnSOD) Levels.

Serum MnSOD levels (ng/mL)	Cases	Controls	OR (95% CI)
	
	N (%)	N (%)	
0-131.6	63 (22.9)	68 (24.7)	1.00 (reference)
131.7-153.8	48 (17.5)	69 (25.1)	0.85 (0.43-1.66)
153.9-202.0	82 (29.8)	69 (25.1)	1.67 (0.89-3.16)
202.1-	82 (29.8)	69 (25.1)	1.54 (0.79-3.01)
			p for trend= 0.06

CI: confidence interval.

Adjusted for pack-years of smoking and *Helicobacter pylori* infection.

Table 4 shows mean values of MnSOD in gastric cancer patients according to clinicopathological findings. Overall, there were no statistically significant differences between the two groups categorized by variables such as disease stage, histological type lymph invasion, lymph node metastasis, liver metastasis, and distant metastasis. We also examined serum MnSOD levels according to site classification, and found no significant differences among the upper, middle, and lower third part (data not shown).

**Table 4.** Mean values of serum manganese superoxide dismutase (MnSOD) in patients with gastric cancer according to clinicopathological findings.

Variables	N	MnSOD(ng/mL)	P value
Stage			
Early	123	176.9± 70.2	0.45
Advance	151	178.9± 98.5	

Histological type			
Intestinal	120	186.8± 103.3	0.23
Diffuse	155	170.1± 72.1	

Lymph invasion			
No	97	172.8± 67.2	0.48
Yes	114	192.5± 104.5	

Venous invasion			
No	155	176.7± 69.7	0.57
Yes	55	203.9± 129.4	

Disseminated to peritoneal			
No	242	178.5± 89.8	0.68
Yes	26	178.4± 64.8	

Lymph node metastasis			
Negative	165	177.1± 68.2	0.73
Postive	97	184.0± 113.8	

Liver metastasis			
No	264	174.2± 70.1	0.008
Yes	4	467.0± 373.5	

Distant metastasis			
No	258	179.9± 87.0	0.23
Yes	3	236.3± 87.2	

Mean ± standard deviation.

## DISCUSSION

Very few studies have examined the role of serum MnSOD levels in the etiology of gastric cancer. Only one earlier study measured serum MnSOD levels in a small number of patients with gastric cancer.^6^ In that study, patients with gastric cancer had relatively higher levels of MnSOD than healthy individuals. Our study, which was based on a relatively large number, showed a statistically marginal increase in serum MnSOD levels among gastric cancer patients compared with matched controls. The mean of serum MnSOD levels among our controls, however, was higher than that in controls in that previous study.^6^ We consider that the differences in the kit used, the characteristics of the study participants, and the condition of serum preservation may have contributed to the variation in serum MnSOD levels. However, in another study that compared serum MnSOD levels in patients with malignant melanoma to normal controls, the mean level was 126 ± 34 ng/mL for 11 normal controls, which approximated the result of our study.^7^

We found that people with serum levels of MnSOD in the highest quartile had a 1.5-fold increased risk of gastric cancer compared with those in the lowest quartile; however, the association was statistically insignificant. This finding should be interpreted cautiously as elevated serum MnSOD may be a result of the malignancy. However, we consider that this possibility was minimal since serum MnSOD levels did not differ significantly between patients with early and advanced gastric cancer and the ORs were also similar for these two groups (data not shown). Additional prospective studies are needed to investigate the causal link between serum MnSOD and gastric cancer.

The mechanisms linking serum MnSOD levels and gastric cancer risk are plausible but merit further exploration. First, epidemiologic studies have suggested that gastric cancer may be caused by reactive oxygen species and dietary antioxidant such as vitamin C may be protective against gastric cancer.^8^^,^^9^ The production of reactive oxygen species was increased in the mucosa of persons with *H. pylori* infection.^10^ Infection with *H. pylori* was associated with an abundant inflammatory response, and could increase the susceptibility of epithelial cells to reactive oxygen species-associated cell injury.^11^ Although we did not observe a significant association between *H. pylori* infection and serum MnSOD levels in our study, experiments by Smoot et al. showed that MnSOD concentrations were significantly increased after exposure of gastric cell line to both cagA- and cagA+ *H. pylori* strains.^11^ Moreover, the generation of reactive oxygen species has been detected in various cells stimulated with growth factors and cytokines, including epidermial growth factor,^12^ tumor necrosis factor-*α*,^13^ and interleukin 1,^14^ which were also thought to cause the increased level of MnSOD. In another study, we have observed a significant increase in serum tumor necrosis factor -*α* levels among patients with gastric cancer (data not shown). Thus, inflammation in the stomach may contribute to high levels of MnSOD. The increased serum MnSOD levels may be a host response to the increased production of reactive oxygen species, which may be derived from ingested food, inflammatory response or cigarette smoking.

Second, the important role of the oxidant-antioxidant balance has been suggested in cancer development.^1^ MnSOD plays an essential role in the conversion of superoxide anion into hydrogen peroxide (H_2_O_2_) in the mitochondrial matrix, and catalase and GPx are also important enzymes which converts H_2_O_2_ to water and oxygen. Decreased catalase activity in tumor cells may lead to the accumulation of H_2_O_2_, which causes DNA damage and/or cell death.^15^ High levels of MnSOD with decreased catalase may create an antiapoptotic intracellular environment which is especially susceptible to increased frequencies of mutation, a situation likely to lead to cell transformation and cancer.^16^ These findings suggest that the disturbance in oxidant-antioxidant balance may promote gastric carcinogenesis.

In our study, no significant differences were noted in serum MnSOD levels between intestinal and diffuse type gastric cancer, and neither were noted between early and advanced gastric cancer. Serum MnSOD levels were also not significantly correlated with other clinicopathological findings. Although one earlier study has shown that a high carcinoma/normal mucosa MnSOD ratio in gastric cancer patients is significantly associated with a poor survival,^17^ the results of our study indicated that serum MnSOD levels may not be useful in predicting the disease progression in gastric cancer patients.

One limitation of this study is that data were not available for gastric ulcers. Gastric ulcer, which may be initiated and aggravated by oxidative stress, is associated with the risk of gastric cancer.^18^ The mechanism underlying the association between *H. pylori* infection, reactive oxygen species, gastric ulcer and gastric cancer are intriguing and will be an important subject for future investigations.

In conclusion, our study did not show that serum MnSOD levels are significantly associated with increased risk of gastric cancer, although a weak association may exist between them. Prospective studies are needed to establish the role of serum MnSOD in the development of gastric cancer.

## References

[1] Oberley TD Oxidative damage and cancer. Am J Pathol 2002; 160: 403-8. 11839558 10.1016/S0002-9440(10)64857-2 PMC1850635

[2] Taniguchi N Clinical significances of superoxide dismutases: changes in aging, diabetes, ischemia, and cancer. Adv Clin Chem 1992; 29: 1-59. 1585847 10.1016/s0065-2423(08)60221-8

[3] Macmillan-Crow LA, Cruthirds DL Invited review: manganese superoxide dismutase in disease. Free Radic Res 2001; 34: 325-36. 11328670 10.1080/10715760100300281

[4] Van Driel BE, Lyon H, Hoogenraad DC, Anten S, Hansen U, Van Noorden CJ Expression of CuZn- and Mn-superoxide dismutase in human colorectal neoplasms. Free Radical Bio Med 1997; 23: 435-44. 9214580 10.1016/s0891-5849(97)00102-0

[5] Japanese Gastric Cancer Association Japanese classification of gastric carcinoma. 2nd English Edition. Gastr Cancer 1998;1:10-24. 10.1007/s101209800016 11957040

[6] Kawaguchi T, Suzuki K, Matsuda Y, Nishimura T, Uda T, Ono M, Serum-manganese-superoxide dismutase: normal values and increased levels in patients with acute myocardial infarction and several malignant diseases determined by an enzyme-linked immunosorbent assay using a monoclonal antibody. J Immunol Methods 1990; 127: 249-54. 2313102 10.1016/0022-1759(90)90075-7

[7] Schadendorf D, Zuberbier T, Diehl S, Schadendorf C, Czarnetzki BM Serum manganese superoxide dismutase is a new tumour marker for malignant melanoma. Melanoma Res 1995; 5: 351-3. 8541726 10.1097/00008390-199510000-00008

[8] Hansson LE, Nyren O, Bergstrom R, Wolk A, Lindgren A, Baron J, Nutrients and gastric cancer risk. A population-based case-control study in Sweden. In J Cancer 1994; 57: 638-44. 10.1002/ijc.2910570505 8194870

[9] Graham S, Haughey B, Marshall J, Brasure J, Zielezny M, Freudenheim J, Diet in the epidemiology of gastric cancer. Nutr Cancer 1990; 13: 19-34. 2300492 10.1080/01635589009514042

[10] Zhang Q, Dawodu JB, Etolhi G, Husain A, Gemmell CG, Russel RI Relationship between the mucosal production of reactive oxygen radicals and density of Helicobacter pylori in patients with duodenal ulcer. Eur J Gastroenterol Hepatol 1997; 9: 261-5. 9096427 10.1097/00042737-199703000-00008

[11] Smoot DT, Elliott TB, Verspaget HW, Jones D, Allen CR, Veron KG, Influence of Helicobacter pylori on reactive oxygen-induced gastric epithelial cell injury. Carcinogenesis 2000; 21: 2091-5. 11062173 10.1093/carcin/21.11.2091

[12] Li X, Nagayasu H, Hamada J, Hosokawa M, Takeichi N Enhancement of tumorigenicity and invasion capacity of rat mammary adenocarcinoma cells by epidermal growth factor and transforming growth factor-beta. Jpn J Cancer Res 1993; 84: 1145-9. 8276719 10.1111/j.1349-7006.1993.tb02814.x PMC5919095

[13] Lo YY, Cruz TF Involvement of reactive oxygen species in cytokine and growth factor induction of c-fos expression in chondrocytes. J Biol Chem 1995; 270 :11727-30. 7744816 10.1074/jbc.270.20.11727

[14] Meier B, Radeke HH, Selle S, Younes M, Sies H, Resch K, Human fibroblasts release reactive oxygen species in response to interleukin-1 or tumour necrosis factor-alpha. Biochem J 1989; 263: 539-45. 2556998 10.1042/bj2630539 PMC1133461

[15] Islam KN, Kayanoki Y, Kaneto H, Suzuki K, Asahi M, Fujii J, TGF-*β*_1_ triggers oxidative modifications and enhances apoptosis in HIT cells through accumulation of reactive oxygen species by suppression of catalase and glutathione peroxidase. Free Radic Biol Med 1997; 90: 1007-17. 10.1016/s0891-5849(96)00493-5 9034240

[16] Chung-man Ho J, Zheng S, Comhair SA, Farver C, Erzurum SC Differential expression of manganese superoxide dismutase and catalase in lung cancer. Cancer Res 2001; 61: 8578-85. 11731445

[17] Janssen AM, Bosman CB, van Duijn W, Oostendorp-van de Ruit MM, Kubben FJ, Griffioen G, Superoxide dismutases in gastric and esophageal cancer and the prognostic impact in gastric cancer. Clin Cancer Res 2000; 6: 3183-92. 10955802

[18] Kwiecien S, Brzozowski T, Konturek PC, Pawlik MW, Pawlik WW, Kwiecien N, The role of reactive oxygen species and capsaicin-sensitive sensory nerves in the patho-mechanisms of gastric ulcers induced by stress. J Physiol Pharmacol 2003; 54: 423-37. 14566080

